# The influence of *APOE*^*ε4*^ on the pTau interactome in sporadic Alzheimer’s disease

**DOI:** 10.1007/s00401-024-02744-8

**Published:** 2024-05-21

**Authors:** Manon Thierry, Jackeline Ponce, Mitchell Martà-Ariza, Manor Askenazi, Arline Faustin, Dominique Leitner, Geoffrey Pires, Evgeny Kanshin, Eleanor Drummond, Beatrix Ueberheide, Thomas Wisniewski

**Affiliations:** 1 Department of Neurology, Center for Cognitive Neurology, Grossman School of Medicine, New York University, Science Building, Rm 1023J, 435 East 30th Street, New York, NY USA; 2 Department of Biochemistry and Molecular Pharmacology, Proteomics Laboratory, Grossman School of Medicine, New York University, New York, NY USA; 3 Institut de Neurociències, Universitat Autònoma de Barcelona, Barcelona, Spain; 4 Biomedical Hosting LLC, Arlington, MA USA; 5 Department of Neurology, Comprehensive Epilepsy Center, Grossman School of Medicine, New York University, New York, NY USA; 6 Brain and Mind Centre, School of Medical Science, University of Sydney, Sydney, Australia; 7 Departments of Pathology and Psychiatry, Grossman School of Medicine, New York University, Science Building, Rm 1017, 435 East 30 Street, New York, NY 10016 USA

**Keywords:** Alzheimer, Tau, ApoE, Interactome, Proteomics, Neuropathology

## Abstract

*APOE*^*ε4*^ is the major genetic risk factor for sporadic Alzheimer’s disease (AD). Although *APOE*^*ε4*^ is known to promote Aβ pathology, recent data also support an effect of *APOE* polymorphism on phosphorylated Tau (pTau) pathology. To elucidate these potential effects, the pTau interactome was analyzed across *APOE* genotypes in the frontal cortex of 10 advanced AD cases (*n* = 5 *APOE*^ε3/ε3^ and *n* = 5 *APOE*^*ε4/ε4*^), using a combination of anti-pTau pS396/pS404 (PHF1) immunoprecipitation (IP) and mass spectrometry (MS). This proteomic approach was complemented by an analysis of anti-pTau PHF1 and anti-Aβ 4G8 immunohistochemistry, performed in the frontal cortex of 21 advanced AD cases (*n* = 11 *APOE*^ε3/ε3^ and *n* = 10 *APOE*^*ε4/ε4*^). Our dataset includes 1130 and 1330 proteins enriched in IP_PHF1_ samples from *APOE*^ε3/ε3^ and *APOE*^*ε4/ε4*^ groups (fold change ≥ 1.50, IP_PHF1_ *vs* IP_IgG ctrl_). We identified 80 and 68 proteins as probable pTau interactors in *APOE*^ε3/ε3^ and *APOE*^*ε4/ε4*^ groups, respectively (SAINT score ≥ 0.80; false discovery rate (FDR) ≤ 5%). A total of 47/80 proteins were identified as more likely to interact with pTau in *APOE*^*ε3/ε3*^* vs APOE*^*ε4/ε4*^ cases. Functional enrichment analyses showed that they were significantly associated with the nucleoplasm compartment and involved in RNA processing. In contrast, 35/68 proteins were identified as more likely to interact with pTau in *APOE*^*ε4/ε4*^* vs APOE*^*ε3/ε3*^ cases. They were significantly associated with the synaptic compartment and involved in cellular transport. A characterization of Tau pathology in the frontal cortex showed a higher density of plaque-associated neuritic crowns, made of dystrophic axons and synapses, in *APOE*^*ε4*^ carriers. Cerebral amyloid angiopathy was more frequent and severe in *APOE*^*ε4/ε4*^ cases. Our study supports an influence of *APOE* genotype on pTau-subcellular location in AD. These results suggest a facilitation of pTau progression to Aβ-affected brain regions in *APOE*^*ε4*^ carriers, paving the way to the identification of new therapeutic targets.

**Supplementary Information:**

The online version contains supplementary material available at 10.1007/s00401-024-02744-8.

## Introduction

Alzheimer’s disease (AD) is characterized by the extracellular deposition and self-aggregation of β-amyloid peptides (Aβ) into various types of Aβ deposits [22, 34], along with the intraneuronal accumulation and self-assembly of abnormally phosphorylated Tau proteins (pTau) into neurofibrillary tangles [14, 36, 37]. These neuropathological lesions affect successively different regions of the brain, following distinct stereotypical sequences described by the five Thal phases (0–5 for Aβ pathology [82]) and the six Braak stages (0-VI for Tau pathology [12]). A polymorphism of the *apolipoprotein E* gene (*APOE*) is the major genetic risk factor associated with sporadic AD. In comparison with the common *APOE*^*ε3*^ allele, the *APOE*^*ε4*^ allele is associated with an increased risk and an earlier onset for AD, in a dose-dependent manner (Odds Ratio *APOE*^*ε4/ε4*^ = 14.2; www.alzgene.org; [19, 77]). In contrast, the rare *APOE*^*ε2*^ allele confers a protection against the development of AD (Odds Ratio *APOE*^*ε2/ε2*^ = 0.7; www.alzgene.org; [18]). In the brain, Apolipoprotein E (ApoE) is a glycoprotein predominantly secreted by astrocytes under physiologic conditions [11] and is involved in phospholipid and cholesterol transport: the C-terminus domain of ApoE binds with phospholipid packed into HDL-like particles conveying cholesterol [30, 68], while the N-terminus domain interacts with receptors of the LDLR family [41, 66].

ApoE is colocalized with parenchymal Aβ deposits in AD, as well as with vascular Aβ accumulation in a context of cerebral amyloid angiopathy (CAA) [57, 91]. The presence of the *APOE*^*ε4*^ allele is strongly associated with an exacerbation of Aβ pathology [70]. Experimental data confirm the influence of *APOE* expression on Aβ accumulation [6, 31], unravelling a differential effect of ApoE isoforms on Aβ clearance impairment, aggregation and fibrillation (ApoE2 < ApoE3 < ApoE4) [15, 23, 39, 89]. In addition to these established effects of ApoE on Aβ pathology, emerging evidence suggests that ApoE also plays an important role in Tau pathology. The neuroanatomical progression of Tau pathology follows the expression gradients of *APOE* [54]. Furthermore, an exceptional resistance to an autosomal dominant familial AD mutation, associated with a massive parenchymal Aβ deposition but a surprisingly discrete Tau pathology, was recently attributed to the co-occurrence of a protective mutation in the *APOE*^*ε3*^ sequence [1, 73]. Although ApoE is known to be colocalized with pTau within neurofibrillary tangles, their relationship remains elusive [57, 76]. In vivo experiments support an Aβ-independent effect of *APOE*^*ε4*^ on pTau accumulation [44, 74], which may involve a promotion of Tau phosphorylation [13, 69] or a disruption of cholesterol metabolism and lysosomal functions [48]. Further investigations are needed in the AD brain to understand how *APOE* impacts Tau pathology, which correlates better than Aβ deposition with the cognitive status of AD cases [58].

The development of localized proteomics on *post-mortem* human brains, by our group, identified de novo proteins associated with Tau pathology [25, 26, 63, 64]. More particularly, the combination of anti-pTau pS396/pS404 immunoprecipitation with downstream proteomic analysis allowed the identification of proteins that specifically interact with pathologic pTau species involved in AD pathology [26]. In contrast, similar approaches focused on proteins interacting with total Tau, without discriminating its physiologic and pathologic forms [4, 9, 40, 49, 51, 53, 85, 87]. In this study, we took advantage of our unbiased strategy to fully uncover the effects of the AD risk factor *APOE*^*ε4*^ on pTau metabolism: we combined anti-pTau immunoprecipitation with MS to map out, for the very first time, the pTau interactome in the AD brain of *APOE*^*ε3*^* vs APOE*^*ε4*^ carriers.

## Materials and methods

### Cases

All procedures were performed under protocols approved by Institutional Review Boards at New York University Alzheimer’s Disease Research Center (NYU ADRC, NY, USA) and Columbia University Alzheimer’s Disease Research Center (CU ADRC, NY, USA). In all cases, written informed consent for research was obtained from the patient or legal guardian, and the material used had appropriate ethical approval for use in this project. All patients’ data and samples were coded and handled according to NIH guidelines to protect patients’ identities. A total of 25 cases of sporadic AD were included in this study. Cases were selected from donated brain tissue collected at the NYU ADRC and CU ADRC, based on their ABC score (A3, B3, C3; [55]), severity of Tau pathology in the frontal cortex and *APOE* genotype. The *APOE*^ε3/ε3^ and *APOE*^ε4/ε4^ groups were matched to the best of our ability in terms of age, sex and co-morbidities, as shown in Table 1. Our inclusion criteria involved indeed the absence of any additional primary tauopathy and of any major co-proteinopathy. For the neuropathological analysis, the presence of a concomitant Lewy Body disease of the amygdala-predominant type was tolerated for *n* = 2 cases per group to increase our number of cases, as this co-pathology is common in the elderly population and because its even distribution among our groups did not impact our comparative study design. Individual case information is detailed in Table 1 (age, sex, *APOE* genotype, *post-mortem* interval (PMI), ABC score, Braak stage, neuropathological findings, technical application).

**Table 1:** Cohort description

Case	Age	Sex	APOE	PMI	Source	ABC score	Braak	Neuropathological findings (other than AD-related changes)	Study
*A/1*	77	F	ε3/ε3	66	NYU ADRC	A3, B3, C3	VI	Hippocampal sclerosis	P, H
*B/2*	90	F	ε3/ε3	21	NYU ADRC	A3, B3, C3	VI	CAA and Binswanger’s disease	P, H
*C/3*	67	M	ε3/ε3	< 48	NYU ADRC	A3, B3, C3	VI	CAA	P, H
*D/4*	83	M	ε3/ε3	142	NYU ADRC	A3, B3, C3	VI	CAA, Binswanger’s disease and hemorrhages	P
*E/5*	85	F	ε3/ε3	< 24	NYU ADRC	A3, B3, C3	VI	CAA and Binswanger’s disease	P
*F*	91	F	ε3/ε3	< 48	NYU ADRC	A3, B3, C3	VI	Hippocampal sclerosis and Binswanger’s disease	H
*G*	87	F	ε3/ε3	26	NYU ADRC	A3, B3, C3	VI	Binswanger’s disease and chronic ischemia (insula)	H
*H*	73	M	ε3/ε3	< 24	NYU ADRC	A3, B3, C3	VI	CAA and Binswanger’s disease	H
*I*	87	F	ε3/ε3	10	CU ADRC	A3, B3, C3	VI	Subdural hematoma (parieto-occipital, left), meningioma psammomatous clival (left) and athero-arteriolosclerosis	H
*J*	83	F	ε3/ε3	18	CU ADRC	A3, B3, C3	VI	Infarct (putamen—posterior limb of internal capsule—body of the caudate), status cribrosus (lenticular nucleus, thalamus), athero-arteriolosclerosis and intracortical telangiectasia (superior parietal lobule, left)	H
*K*	77	F	ε3/ε3	11	CU ADRC	A3, B3, C3	VI	Vascular brain injury, athero-arteriolosclerosis, CAA and synechia (hippocampo-ventricular, left)	H
*L*	72	M	ε3/ε3	12	CU ADRC	A3, B3, C3	VI	Lewy body disease (amygdala predominant), vascular brain injury, athero-arteriolosclerosis and CAA	H
*M*	70	F	ε3/ε3	21	CU ADRC	A3, B3, C3	VI	Lewy body disease (amygdala predominant), vascular brain injury, athero-arteriolosclerosis, CAA, ferro-calcic vasculopathy (pallidum), hypoxic-ischemic encephalopathy and atrophy of the optic nerve (left > right) and of the lateral geniculate body	H
*N/6*	91	F	ε4/ε4	21	NYU ADRC	A3, B3, C3	VI	CAA and Binswanger’s disease	P
*O/7*	81	F	ε4/ε4	< 48	NYU ADRC	A3, B3, C3	VI	CAA, Binswanger’s disease and hippocampal sclerosis (with TDP43 inclusions)	P, H
*P/8*	79	F	ε4/ε4	< 72	NYU ADRC	A3, B3, C3	VI	CAA and arachnoid cyst	P
*Q/9*	73	F	ε4/ε4	< 48	NYU ADRC	A3, B3, C3	VI	CAA and Binswanger’s disease	P, H
*R/10*	69	M	ε4/ε4	< 24	NYU ADRC	A3, B3, C3	VI	CAA	P, H
*S*	68	F	ε4/ε4	29	NYU ADRC	A3, B3, C3	VI	CAA, Binswanger’s disease and atrophy of the grey and white matter	H
*T*	63	M	ε4/ε4	64	NYU ADRC	A3, B3, C3	VI	N/A	H
*U*	90	F	ε4/ε4	39	CU ADRC	A3, B3, C3	VI	Infarcts (putamen — external capsule, right; cortico-subcortical, angular parietal gyrus, left), hemorrhage (frontal cortex, right), status cribrosus (striatum), athero-arteriolosclerosis and CAA	H
*V*	81	F	ε4/ε4	14	CU ADRC	A3, B3, C3	VI	Lewy body disease (amygdala predominant), metastatic carcinoma, hemorrhage, athero-arteriolosclerosis, CAA and synechia (hippocampo-ventricular, bilateral)	H
*W*	75	F	ε4/ε4	14	CU ADRC	A3, B3, C3	VI	Status cribrosus (striatum), synechia (hippocampo-ventricular, bilateral), athero-arteriolosclerosis and CAA	H
*X*	77	F	ε4/ε4	22	CU ADRC	A3, B3, C3	VI	Lewy body disease (amygdala predominant), vascular brain injury, athero-arteriolosclerosis, occlusive clot in small leptomeninges artery (superior parietal lobule, right), CAA with dyshoric changes (calcarine cortex), ferro-calcic vasculopathy (globus pallidus), small aneurysms (Willis circle)	H
*Y*	81	M	ε4/ε4	17	CU ADRC	A3, B3, C3	VI	Arteriolosclerosis and CAA	H

The 25 cases of neuropathologically confirmed sporadic AD included in our study are listed in this table. The latter discloses their age at death, sex, APOE genotype (APOE), PMI in hours, source, ABC score of AD-related pathologic changes [55], Braak stage [12] and summary of any other neuropathological findings. A letter was attributed to all 25 disidentified cases (A-Y), associated with an additional number for the 10 cases included in the proteomic study to match the proteomic dataset annotations (1–10). The last column detailed the study in which the case was included (“P” for proteomics when frozen tissue was available; “H” for histology when formalin-fixed paraffin-embedded tissue was available). *AD* Alzheimer’s disease, *CAA* Cerebral Amyloid Angiopathy, *CU ADRC* Columbia University Alzheimer’s Disease Research Center, *NYU ADRC* New York University Alzheimer’s Disease Research Center

### Genotyping

*APOE* genotypes were provided by the NYU ADRC and CUMC brain banks for 15 out of 25 cases. For cases 1, 2, 4, 6, 7, 8, 14, 15, 16 and 18, *APOE* genotyping was performed as previously described [24]. A fragment of frozen frontal cortex was dissected (~ 25 mg), then collected into a 1.5 mL tube using single-use consumables in DNA-free experimental conditions. DNA was isolated using the DNeasy Blood & Tissue kit following the manufacturer’s instructions (#69,504, Qiagen). A single endpoint PCR was performed in a total volume of 25 µl containing 0.2 µM of each custom primer (Forward primer 5′ AGCCCTTCTCCCCGCCTCCCACTGT 3′; reverse primer 5′ CTCCGCCACCTGCTCCTTCACCTCG 3′; Millipore Sigma), 10 µl of DreamTaq Green PCR Master Mix 2X (#K1081, Thermo Scientific) and 4.2 µl of Betaine (#B0300, Millipore Sigma). Cycling conditions were set as follows: 98 °C for 4 min, 35 cycles at 98 °C/10 s, 63 °C/45 s and 72 °C/1 min 10 s, followed by 72 °C for 10 min. Unpurified PCR products were submitted to Genewiz for Sanger sequencing and the sequences were analyzed using the SnapGene 5.3.1 software.

### Homogenization

Ten cases of sporadic AD were used for proteomic analysis (Table 1). The grey matter was dissected from the frontal cortex of archived fresh frozen human tissue samples stored at  – 80 °C (~ 0.25 g per sample). Cortical tissue was homogenized as previously described [26]. Frozen tissue was enveloped into aluminum foil and pulverized on dry ice using a hammer. The powder was collected into a Dounce homogenizer, then homogenized on ice with ~ 25 strokes in a low salt homogenization buffer (50 mM HEPES pH 7.0, 250 mM sucrose, 1 mM EDTA) with inhibitors of proteases (cOmplete ULTRA Tablets, Mini, EDTA-free; #5,892,791,001, Millipore Sigma) and phosphatases (PhosphoSTOP EASYpack; #4,906,845,001, Millipore Sigma). The total protein concentration of homogenates was assessed with the Micro BCA Protein Assay Kit, following the supplier’s guidelines (#23,235, Thermo Scientific). Samples were stored at  – 80 °C until use.

### Immunoprecipitation

For each case, two immunoprecipitation products were obtained: the first using the mouse antibody anti-pTau pS396/pS404 (PHF1, provided by Dr. Peter Davies, Albert Einstein University, NY, USA [35]) to enrich pTau and its binding partners, the second using a mouse isotype antibody to control non-specific binding (#400,202, BioLegend). As a result, 20 separate IP products were individually analyzed for proteomics. Each IP product required a total of six-reaction mixes to collect enough material for downstream biochemistry and proteomics analyses, using the Dynabeads Protein G Immunoprecipitation Kit and following the supplier’s guidelines with minor adjustments (#10007D, Thermo Scientific). For each reaction mix, brain homogenate (300 μg total proteins/mix) and antibodies (4 μg antibodies/mix) were incubated overnight at 4 °C with over-end rotation to allow antigen–antibody interaction. The next day, the samples containing antigen–antibody complexes were mixed with Dynabeads (1.5 mg/mix), then incubated overnight at 4 °C with over-end rotation. The antigen–antibody-Dynabeads complexes were recovered and washed using a DynaMag-2 magnet (#12321D, Thermo Scientific), then resuspended in 100 μl of phosphate buffered saline at pH 7.4. The six-reaction mixes were pooled into a new tube (600 μL total), to avoid the co-elution of proteins bound to the tube wall. A total of 500 μL of the IP product was kept at 4 °C until proteomics analysis. The remaining 100 μL were eluted by capturing the antigen–antibody-Dynabeads complexes with the magnet, before incubating the beads in 20 μL of a denaturing buffer (141 mM Tris base, 106 mM Tris HCl, 2% SDS, 0.51 mM EDTA, pH 8.5; 15 min at 70 °C and 1000 rpm). The eluted fractions were recovered on the magnet and stored at  – 20 °C until analysis.

### Biochemistry analysis

Western blotting was performed to confirm the enrichment of pTau in the IP products. The equivalent of 10% of the IP product submitted to proteomic analyses was mixed with DTT 100 mM and 4X Bolt LDS Sample Buffer (#B0007, Thermo Scientific), then boiled at 98 °C for 5 min. Proteins were resolved on 4–12% Bis–Tris gels (#NP0322BOX, Thermo Scientific), then transferred onto a 0.2 μm nitrocellulose membrane (#1,620,112, BioRad). Membranes were blocked with 5% milk in Tris-buffered saline with 0.1% Tween-20 for 1 h, then probed with an anti-pTau S199/S202 antibody (1:1500; #44-768G, Thermo Scientific) at room temperature for 1 h, before being incubated with an anti-rabbit horseradish peroxidase antibody (1:3000; #NA934, Cytiva). The signal was revealed using the Pierce ECL Western Blotting Substrate (#32,106, Thermo Scientific) and membranes were imaged with the ChemiDoc MP Imaging System (BioRad). Silver staining was conducted to confirm the presence of a sufficient amount of proteins in our samples for proteomics downstream analysis. The equivalent of 5% of the IP product used in proteomics was mixed with DTT 100 mM and 4X Bolt LDS Sample Buffer (#B0007, Thermo Scientific), then boiled at 98 °C for 5 min. Proteins were resolved on 4–12% Bis–Tris gels (#NP0322BOX, Thermo Scientific). The gels were extracted and the proteins were stained using the SilverQuest Silver Staining Kit following the supplier’s guidelines (#LC6070, Thermo Scientific), to confirm the presence of a sufficient amount of proteins for proteomic downstream analyses. Gels were imaged with the ChemiDoc MP Imaging System (BioRad).

### Proteomic analysis

IP products were analyzed by liquid-chromatography and mass spectrometry (LC–MS/MS), as previously detailed with some adjustments [26].

### On-bead digestion and protein extraction

The antigen–antibody-bead complexes were recovered on a magnet then washed twice with ammonium bicarbonate 100 mM. Samples were reduced with DTT 0.2 M at 57 °C for 1 h, then alkylated with iodoacetamide 0.5 M at RT in the dark for 45 min. Sequencing-grade modified trypsin (Promega) was added to the sample for overnight digestion on a shaker at room temperature (300 ng). The next day, samples were acidified to pH 2 using 10% trifluoroacetic acid, then loaded onto equilibrated Ultra-Micro SpinColumns (Harvard Apparatus) using a microcentrifuge, before being rinsed three times with 0.1% TFA. The extracted samples were further washed with 0.5% acetic acid. The peptides were eluted with 40% acetonitrile in 0.5% acetic acid, followed by the addition of 80% acetonitrile in 0.5% acetic acid. Organic solvent was removed using a SpeedVac concentrator, before reconstituting samples in 0.5% acetic acid.

### LC–MS/MS analysis

A total of 1 μg of protein was analyzed for each sample. A liquid chromatography (LC) separation was performed online with MS using the autosampler of an EASY-nLC 1000 (Thermo Scientific). Peptides were gradient-eluted from the column into the Orbitrap Eclipse using an 85 min gradient (Thermo Scientific). Solvent A consisted of 2% acetonitrile in 0.5% acetic acid and solvent B of 80% acetonitrile in 0.5% acetic acid. The gradient was held at 5% solvent B for 5 min, ramped to 35% solvent B in 60 min, to 45% solvent B in 10 min and to 100% solvent B in another 10 min. High-resolution full MS spectra were acquired with a resolution of 120,000, an AGC target of 4e5, a maximum ion time of 50 ms and a scan range of 400 to 1,500 m/z. All MS/MS spectra were recorded in the orbitrap analyzer using the following instrument parameters: resolution of 30,000, AGC target of 2e5, maximum ion time of 200 ms, one microscan, 2 m/z isolation window and NCE of 27.

### Data processing

The MS/MS spectra were searched against the UniProt human database using Sequest within Proteome Discoverer 1.4. The data were filtered to better than 1% peptide and protein FDR searched against a decoy database. Only proteins with at least two different peptides were considered for downstream analysis. To asses if there are any differences in phosphorylation between the *APOE* groups, we analyzed Tau-protein phosphorylation using Byos (ProteinMetrics). The phosphorylation assignment and area integration were manually verified using the Byos interface. The area under the curve for the same peptide, with and without phosphorylation, was integrated and the amount of phosphorylation reported as a percentage. Note: not all phospho-sites have been observed in all samples and their frequency of observation has been highlighted in Fig. 4.

### Data analysis

Data were analyzed using the Significance Analysis of INTeractome express algorithm (SAINT), as previously detailed [26, 78]. All non-human proteins, introduced during sample preparation, were removed from the results. The proteins were ranked by SAINT score and proteins with a SAINT score ≥ 0.80, equivalent to a FDR of ≤ 5%, were considered as pTau interactors and further studied. Three lists of pTau interactors of interest were submitted to and analyzed with STRING 11.5 and Cytoscape 3.9.1, to investigate network functional enrichments based on the Gene Ontology (GO) terms “cellular component” and “biological process”, using the total genome as background and a redundancy cut-off of 0.7: (1) proteins identified as pTau interactors in both *APOE* groups (SAINT score ≥ 0.80 in *APOE*^ε3/ε3^ and *APOE*^*ε4/ε4*^ groups, *n* = 33 proteins), (2) proteins identified as pTau interactors associated with an *APOE*^ε3/ε3^ genotype (SAINT score ≥ 0.80 in *APOE*^ε3/ε3^ cases, SAINT score < 0.80 in *APOE*^*ε4/ε4*^ cases, *n* = 47 proteins) and 3) proteins identified as pTau interactors associated with an *APOE*^*ε4/ε4*^ genotype (SAINT score ≥ 0.80 in *APOE*^*ε4/ε4*^ cases, SAINT score < 0.80 in *APOE*^ε3/ε3^ cases, *n* = 35 proteins). Network images were extracted and enrichment tables were exported then analyzed using Excel and GraphPad Prism 9. 4. 1. In addition, proteins considered as pTau interactors associated with one *APOE* genotype or the other were further compared among *APOE* groups based on their fold change (FC), calculated as follows: FC = [mean #peptide spectral matches (group of interest) + 1]/[mean #peptide spectral matches (group of reference) + 1].

### Data comparison with previous MS-based studies

Our data were systematically compared to previous AD-related proteomic studies, using two complementary approaches. First, the pTau interactors identified here were compared to our previous study using the same anti-pTau pS396/pS404 (PHF1) antibody and a similar experimental strategy, but different AD tissues and a less stringent SAINT score cut-off (SAINT score ≥ 0.65, FDR ≤ 10% [26]). Second, we interrogated our probable pTau interactors using the NeuroPro searchable database v1.12 [3]. NeuroPro is a website that compiled 38 experimental MS-based proteomic datasets designed to assess protein changes occurring specifically in the AD brain. The following filters were applied: conditions “AD” (proteins associated with the AD brain) and “AD/C” (proteins altered in AD *vs* control brains). The resulting tables were exported and analyzed using Excel.

### Immunohistochemistry

Immunohistochemistry was performed on formalin-fixed paraffin-embedded 8 µm-thick sections of frontal cortex. Sections were deparaffinized and rehydrated through a series of xylene and ethanol washes. Antigen retrieval was performed by treatment with 88% formic acid for 7 min, followed by boiling in citrate buffer (10 mM sodium citrate, 0.05% Tween-20; pH 6). Sections were blocked with 10% normal goat serum, then incubated overnight at 4 °C with a primary antibody anti-pTau pS396/pS404 (1:200; mouse antibody PHF1; provided by Dr. Peter Davies, Albert Einstein University, NY, USA) or anti-Aβ 4G8 (1:1000; mouse antibody; #800,711, BioLegend), diluted in 4% normal goat serum. Sections were incubated for 1 h at room temperature with an anti-mouse secondary antibody (1:1000, #BA-2000, Vector Laboratories), revealed with an avidin–biotin complex HRP detection kit (#PK-6100, Vector Laboratories) in combination with a DAB substrate kit (#34,065, Thermo Scientific), counterstained with Mayer’s hematoxylin (#MHS16, Millipore Sigma) and coverslipped (#P36970, Thermo Scientific). This technique was applied on 21 cases (*n* = 11 *APOE*^*ε3/ε3*^ cases, *n* = 10 *APOE*^*ε4/ε4*^ cases; Table 1).

### Immunohistochemistry quantification

Tau pathology was quantified in the frontal cortex on anti-pTau pS396/pS404 PHF1 immunohistochemistry. Slides were scanned at a 40 × magnification with the Aperio VERSA 8 scanner and analyzed with Aperio ImageScope 12.4.3.5008 (Leica Biosystems). For each case, Tau pathology was quantified in the grey matter from three ROIs of 5.0 × 10^6^ µm^2^ (± 0.5 × 10^6^ µm^2^), encompassing all cortical layers and evenly spread over the cortical section. The total burden of PHF1 immunoreactive material was obtained by running the open source “Positive Pixel Count 2004-08-11” algorithm on each ROI, with the color saturation threshold set at 0.2. Raw data were exported on Excel to calculate the averaged percentage of immunopositive pixels out of the total number of pixels per case. Each neuropathological lesion composing Tau pathology was analyzed on their respective ROI: the first ROI was used to count the number of PHF1 positive neuronal profiles (pre-tangles, tangles and ghost tangles) per µm^2^; the second ROI was used to count the number of PHF1 positive neuritic crowns per µm^2^; in the third ROI, the burden of PHF1 positive neuropil threads was evaluated by assessing the averaged percentage of immunopositive pixels out of the total number of pixels from ten sub-ROIs of 1.0 × 10^4^ µm^2^, covering neuropil areas evenly spread within the ROI and devoid of any neuronal profiles or neuritic crowns. Tau pathology was also quantified in the white matter from ten ROIs of 1.0 × 10^5^ µm^2^ to assess the averaged percentage of immunopositive pixels out of the total number of pixels reflecting the burden of axonal threads. Unpaired non-parametric Mann–Whitney tests were performed on GraphPad Prism 9. 4. 1. to compare each of these ratios among *APOE* groups at a risk level of α = 0.05. Aβ pathology was quantified in the frontal cortex on anti-Aβ 4G8 immunohistochemistry. Slides were scanned at a 20 × magnification with the Aperio VERSA 8 scanner and analyzed with Aperio ImageScope 12.4.3.5008 (Leica Biosystems). For each case, Aβ pathology was quantified in the grey matter from three ROIs of 5.0 × 10^6^ µm^2^ (± 0.5 × 10^6^ µm^2^), encompassing all cortical layers and evenly spread over the cortical section. The total burden of 4G8 immunoreactive material was obtained by running the open source “Positive Pixel Count 2004-08-11” algorithm on each ROI, with the color saturation threshold set at 0.1. Raw data were exported on Excel to calculate the averaged percentage of immunopositive pixels out of the total number of pixels per case. CAA was analyzed in the parenchyma of the frontal cortex on anti-Aβ 4G8 immunohistochemistry, by attributing a semi-quantitative score to each case using the following criteria: 0 if none (no Aβ-positive vessel detected), 1 if sparse (< 25% of Aβ-positive vessels), 2 if moderate (about 50% of Aβ-positive vessels), 3 if severe (about 100% of Aβ-positive vessels). The type of CAA was evaluated by attributing a “Type 1” to cases presenting with capillary CAA along with CAA in larger vessels or a “Type 2” to cases presenting with CAA without capillary involvement [80].

## Results

### Proteomic overview

**Fig. 1:**
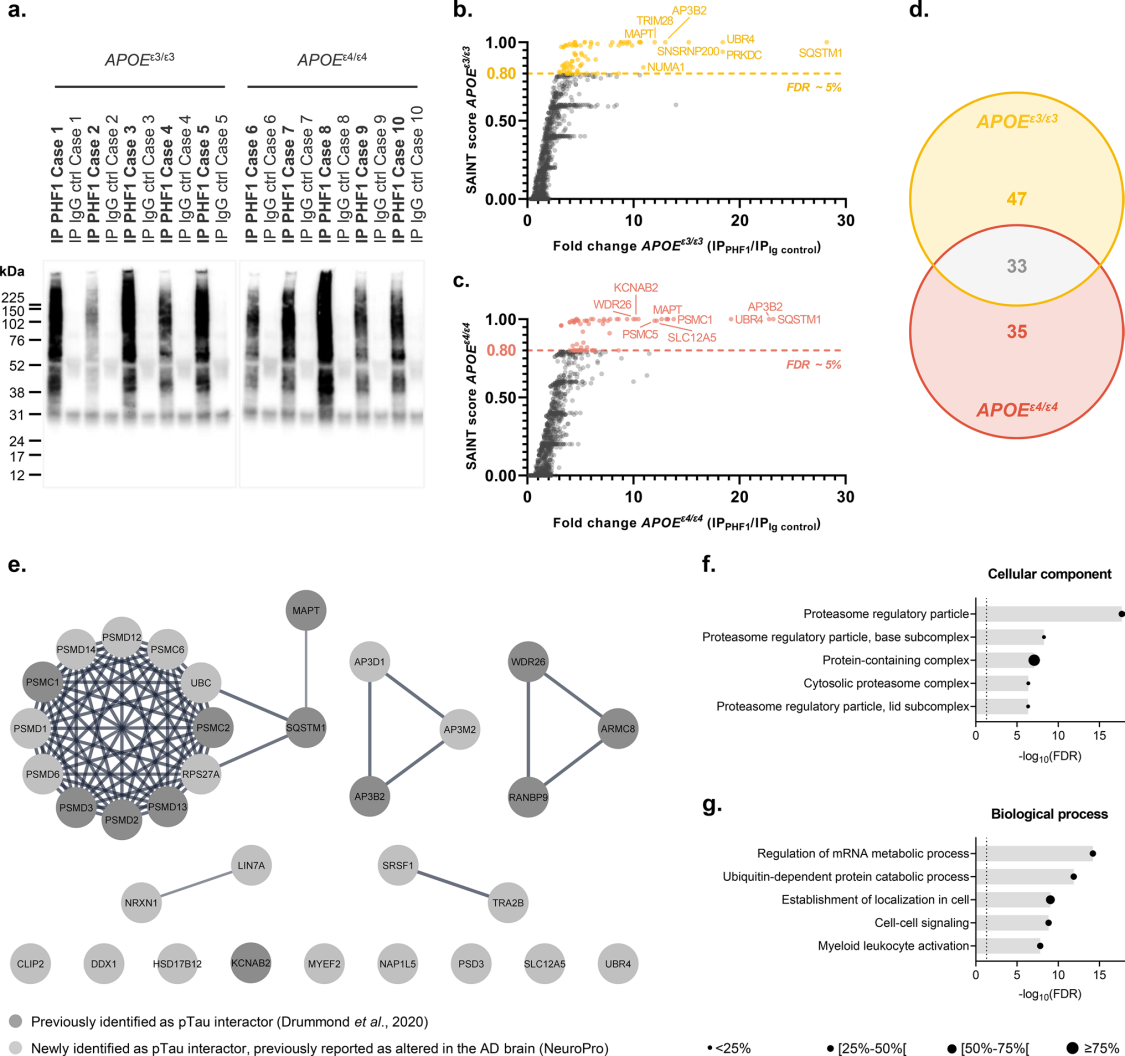
Proteomic overview of the pTau interactome in sporadic AD cases of various *APOE* genotypes. **a** Western blot of the equivalent of 10% of the IP_PHF1_ or IP_IgG ctrl_ products obtained from homogenates of frozen frontal cortex from 10 neuropathologically confirmed AD cases (cases 1–5 of *APOE*^ε3/ε3^ genotype, cases 6–10 of *APOE*^ε4/ε4^ genotype). **b**–**c**. One-sided volcano plots representing, for all proteins identified by LC–MS/MS, their SAINT score as a function of their fold change IP_PHF1_/IP_IgG ctrl_ for the *APOE*^ε3/ε3^ group (**b**) or for the *APOE*^ε4/ε4^ group (**c**). d. Venn diagram representing the 80 and 68 proteins identified as probable pTau interactors in the *APOE*^ε3/ε3^ and *APOE*^ε4/ε4^ groups, respectively; 33 of these pTau interactors were common to both groups (SAINT score ≥ 0.80, FDR ≤ 5%). **e** Network representation of the 33 common pTau interactors. Each protein is represented by its gene ID as a node. The interacting nodes are connected by edges and their thickness indicates the strength of data support with a high confidence interaction score set at 0.7 (STRING). The node color reflects the protein status regarding previous AD-related proteomic studies, as detailed in the legend. **f**–**g**. Description of the functional enrichments associated with the 33 common pTau interactors as a function of –log_10_(FDR), using “genome” as background and a redundancy cut-off of 0.7. The incorporated bubble plots reflect the number of corresponding proteins in the network as a percentage, as detailed in the legend (PPI enrichment *p* value = 1.0 × 10^–16^; STRING and Cytoscape). The top panel details the top 5 GO terms “cellular component” (**f**) while the bottom panel shows the top 5 GO terms “biological process” (**g**). *AD: Alzheimer’s disease*; *IP: Immunoprecipitation; FDR: false discovery rate*

Immunoprecipitated fractions were obtained from frontal cortex homogenates of 10 advanced sporadic AD cases (*n* = 5 *APOE*^ε3/ε3^ and *n* = 5 *APOE*^*ε4/ε4*^ cases), using anti-pTau PHF1 (IP_PHF1_) or control IgG (IP_IgG ctrl_) antibodies. The enrichment of pTau in the IP_PHF1_ products was confirmed by western blot (Fig. 1a). A total of 1130 and 1330 proteins were detected in the IP_PHF1_ samples of the *APOE*^ε3/ε3^ and *APOE*^*ε4/ε4*^ groups (fold change ≥ 1.50, IP_PHF1_ *vs* IP_IgG ctrl_, Online Resource 1). Our dataset was filtered using the probabilistic SAINT score to identify the most likely pTau interactors, leading to the identification of 80 proteins of interest in the *APOE*^ε3/ε3^ group and 68 in the *APOE*^*ε4/ε4*^ group (SAINT scores ≥ 0.80, FDR ≤ 5%). Among these, 33 proteins were common to both *APOE* groups (Fig. 1b–d). They included 12/33 proteins previously identified as pTau interactors by our laboratory: AP3B2, ARMC8, KCNAB2, MAPT, PSMC1, PSMC2, PSMD13, PSMD2, PSMD3, RANBP9, SQSTM1 and WDR26 [26]. The remaining 21/33 proteins were identified as pTau interactors for the first time here, although they have been previously reported to have significantly altered protein levels in AD brain tissue (Fig. 1e and Online Resource 2; [3]). A significant network functional enrichment was associated with the 33 most probable pTau interactors common to *APOE*^ε3/ε3^ and *APOE*^*ε4/ε4*^ cases (PPI enrichment *p* value = 1.0 × 10^–16^; Online Resource 3a): they were predominantly associated with the proteasome system (GO terms “cellular component”, Fig. 1f) and involved in the regulation of mRNA metabolism, ubiquitin-dependent catabolic process or establishment of localization in cell (GO terms “biological process”, Fig. 1g).

### The pTau interactome in sporadic AD cases with an APOE^ε3/ε3^ genotype

**Fig. 2:**
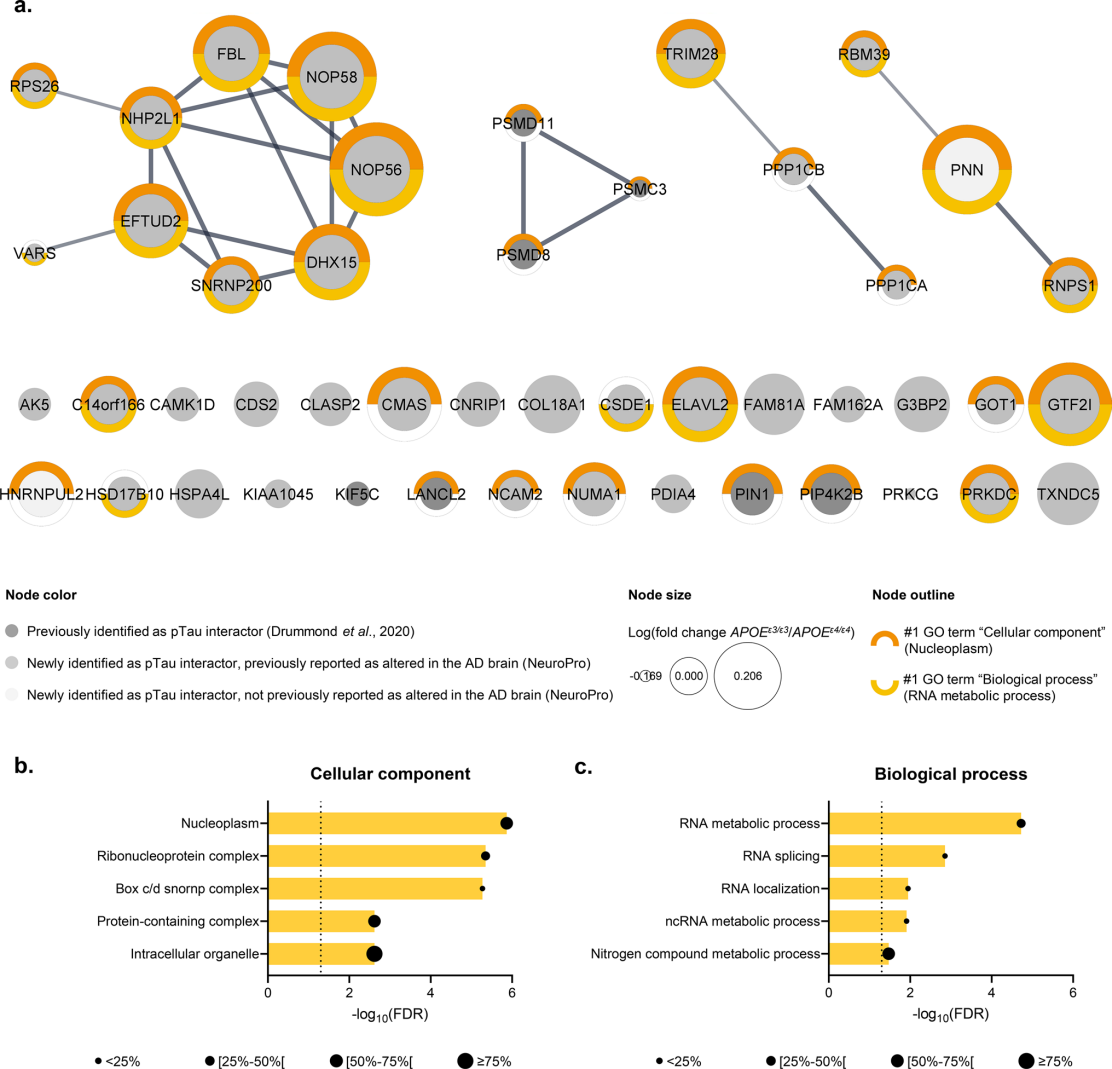
The pTau interactome associated with sporadic AD cases of *APOE*^ε3/ε3^ genotype. **a** Network representation of the 47 proteins identified as probable pTau interactors associated with the *APOE*^ε3/ε3^ group (SAINT score (*APOE*^ε3/ε3^) ≥ 0.80 *vs* SAINT score (*APOE*^ε4/ε4^) < 0.80). Each protein is represented by its gene ID as a node, which size reflects its relative abundance in comparison to the *APOE*^ε4/ε4^ group (log_10_(fold change *APOE*^ε3/ε3^/*APOE*^ε4/ε4^)). The interacting nodes are connected by edges and their thickness indicates the strength of data support with a high confidence interaction score set at 0.7 (STRING). The node color reflects the protein status regarding previous AD-related proteomic studies, as detailed in the legend. These 47 proteins were mainly associated with the nucleoplasm (29/47; #1 GO term “cellular component”, *orange outline*) and involved in RNA metabolic processes (19/47; #1 GO term “biological process”, *yellow outline*). **b**–**c** Description of the functional enrichments associated with these 47 pTau interactors specific to the *APOE*^ε3/ε3^ group as a function of –log_10_(FDR), using “genome” as background and a redundancy cut-off of 0.7. The incorporated bubble plots reflect the number of corresponding proteins in the network as a percentage, as detailed in the legend (PPI enrichment *p* value = 7.1 × 10^–3^; STRING and Cytoscape). The left panel details the top 5 GO terms “cellular component” (**b**), the right panel shows the top 5 GO terms “biological process” (**c**). All terms were ranked according to their FDR, the dashed lines materializing a significance threshold of FDR = 5%. *FDR *false discovery rate

A total of 47 proteins were identified as most likely pTau interactors only for *APOE*^ε3/ε3^ cases (SAINT score (*APOE*^ε3/ε3^) ≥ 0.80, average SAINT score (*APOE*^ε3/ε3^) ± SEM = 0.91 ± 0.01 *vs* SAINT score (*APOE*^ε4/ε4^) < 0.80, average SAINT score (*APOE*^ε4/ε4^) ± SEM = 0.52 ± 0.04). A significant network functional enrichment was attributed to these 47 proteins (PPI enrichment *p* value = 7.1 × 10^–3^; Online Resource 3b). In this network, 7/47 proteins were previously reported as probable pTau interactors by our group: KIF5C, LANCL2, PIN1, PIP4K2B, PSMC3, PSMD8 and PSMD11 [26]. The remaining 40/47 proteins were identified as pTau interactors for the first time here, although they included 38 proteins previously reported to have significantly altered protein levels in AD brain tissue (Fig. 2a and Online Resource 2; [3]). We detected four proteins particularly enriched in *APOE*^ε3/ε3^ cases, in comparison to the *APOE*^ε4/ε4^ group: NOP56, NOP58, PNN, TXNDC5 (fold change ≥ 1.50, IP_PHF1_(*APOE*^ε3/ε3^) *vs* IP_PHF1_(*APOE*^*ε4/ε4*^), ranked by relative abundance). The 47 proteins were predominantly enriched in proteins associated with the nucleoplasm (29/47 proteins; #1 GO term “cellular component”) and involved in RNA metabolic processes (19/47 proteins; #1 GO term “biological process”; Fig. 2a). A detailed analysis of the top significant functional enrichments associated with this network, ranked as per their FDR, emphasized a large predominance of functions associated with RNA binding and processing (Fig. 2b, top 5 GO terms “Cellular component”; Fig. 2c, top 5 GO terms “biological process”).

### The pTau interactome in sporadic AD cases with an APOE^ε4/ε4^ genotype

**Fig. 3:**
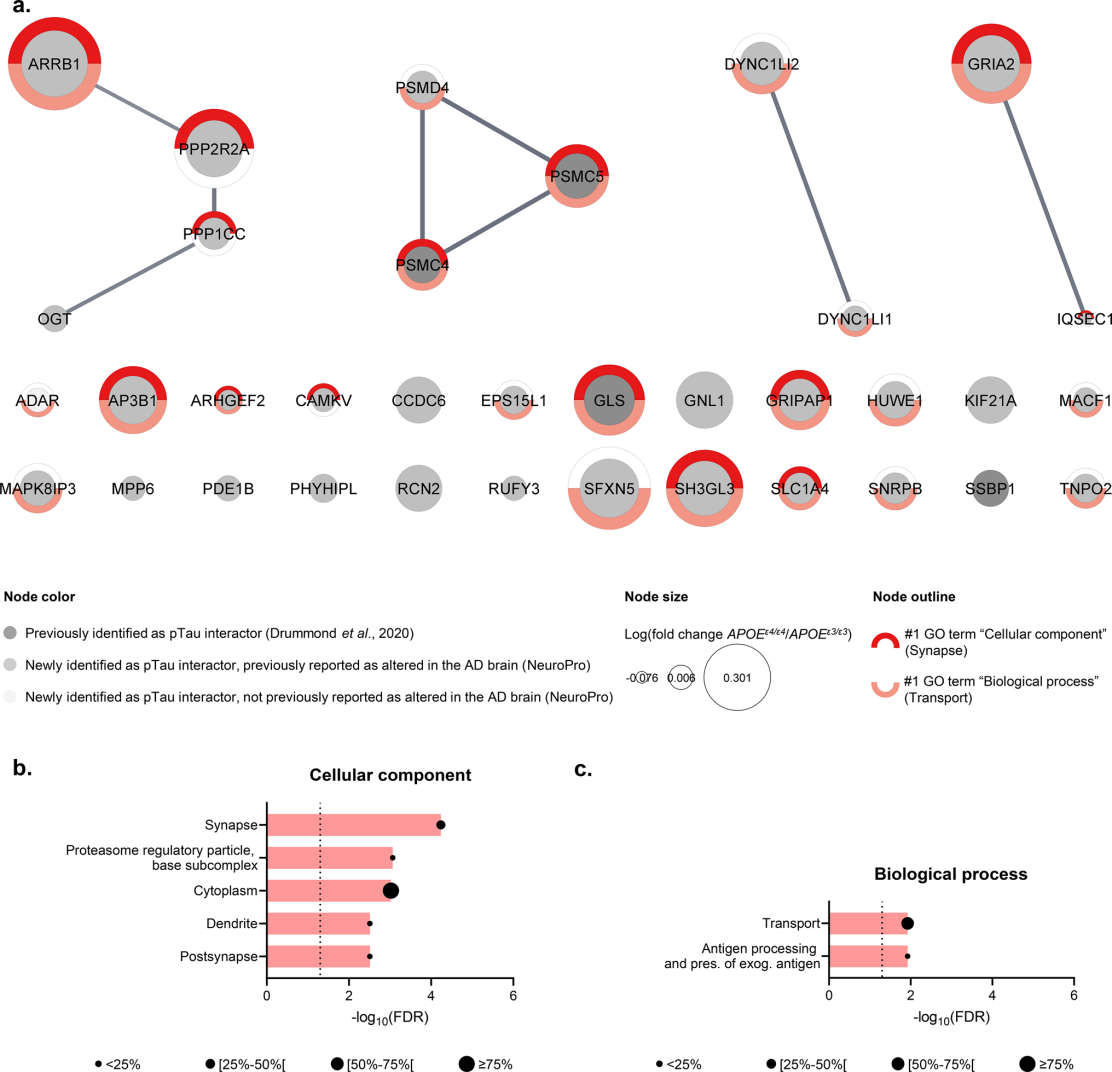
The pTau interactome associated with sporadic AD cases of *APOE*^ε4/ε4^ genotype. **a** Network representation of the 35 proteins identified as probable pTau interactors associated with the *APOE*^ε4/ε4^ group (*APOE*^ε4/ε4^) ≥ 0.80 *vs* SAINT score (*APOE*^ε3/ε3^) < 0.80). Each protein is represented by its gene ID as a node, which size reflects its relative abundance in comparison to the *APOE*^ε3/ε3^ group (log_10_(fold change *APOE*^ε4/ε4^/*APOE*^ε3/ε3^)). The interacting nodes are connected by edges and their thickness indicates the strength of data support with a high confidence interaction score set at 0.7 (STRING). The node color reflects the protein status regarding previous AD-related proteomic studies, as detailed in the legend. These 35 proteins were mainly associated with the synapse (14/35; #1 GO term “cellular component”, *red outline*) and involved in transport pathways (21/35; #1 GO term “biological process”, *pink outline*). **b**–**c**. Description of the functional enrichments associated with these 35 pTau interactors specific to *APOE*^ε4/ε4^ cases as a function of –log_10_(FDR), using the total genome as background and a redundancy cut-off of 0.7. The incorporated bubble plots reflect the number of corresponding proteins in the network as a percentage, as detailed in the legend (PPI enrichment *p* value = 2.4 × 10^–2^; STRING and Cytoscape). The left panel details the top 5 GO terms “cellular component” (**b**), the right panel shows the top five GO terms “biological process” (**c**). All terms were ranked according to their FDR, the dashed lines materializing a significance threshold of FDR = 5%. *FDR* false discovery rate

By analogy, 35 proteins were identified as most likely pTau interactors only for *APOE*^ε4/ε4^ cases (SAINT score (*APOE*^ε4/ε4^) ≥ 0.80, average SAINT score (*APOE*^ε4/ε4^) ± SEM = 0.88 ± 0.02 *vs* SAINT score (*APOE*^ε3/ε3^) < 0.80, average SAINT score (*APOE*^ε3/ε3^) ± SEM = 0.60 ± 0.02). A significant network functional enrichment was associated with these 35 proteins (PPI enrichment *p* value = 2.4 × 10^–3^; Online Resource 3c). In this network, 4/35 proteins were previously reported as probable pTau interactors by our laboratory: GLS, PSMC4, PSMC5 and SSBP1 [26]. The remaining 31/35 proteins corresponded to pTau interactors identified for the first time here, although they included 30 proteins previously reported to have significantly altered protein levels in AD brain tissue (Fig. 3a and Online Resource 2; [3]). We identified eight proteins particularly enriched in *APOE*^ε4/ε4^ cases, in comparison to the *APOE*^ε3/ε3^ group: ARRB1, SFXN5, GNL1, GRIA2, PP2R2A, SH3GL3, GLS, AP3B1 (fold change ≥ 1.50, IP_PHF1_ (*APOE*^ε4/ε4^) *vs* IP_PHF1_ (*APOE*^*ε3/ε3*^), ranked by relative abundance). This network of 35 proteins was predominantly associated with the synaptic compartment (14/35; #1 GO term “cellular component”) and involved in intracellular transport (21/35; #1 GO term “biological process”; Fig. 3a). A detailed analysis of the top significant functional enrichments associated with this network, ranked by FDR, depicted a majority of functions associated with synaptic transmission and cellular trafficking (Fig. 3b, top 5 GO terms “cellular component”; Fig. 3c, top 5 GO terms “biological process”).

### Tau-phosphorylation landscape across APOE genotypes

**Fig. 4:**
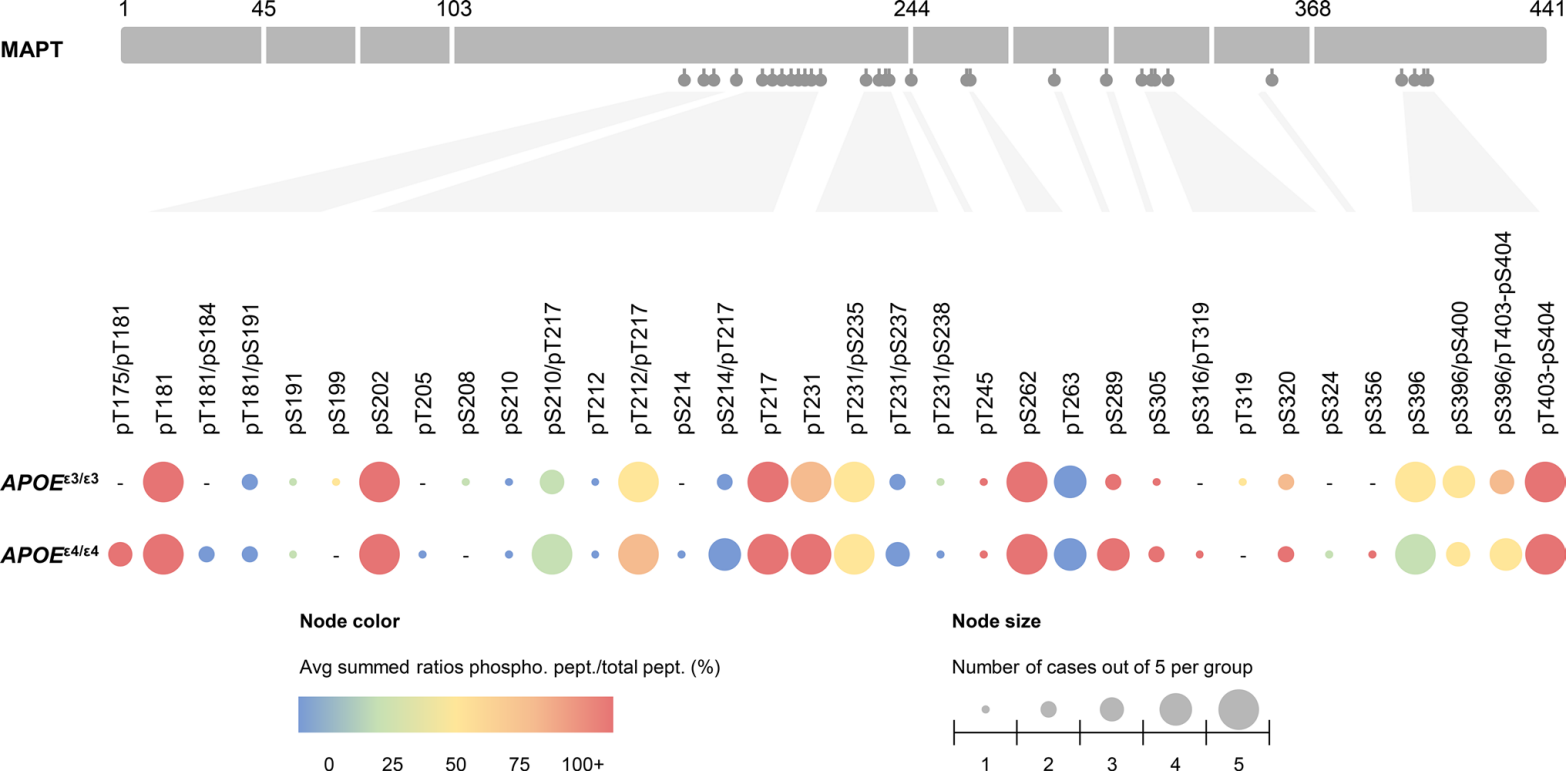
The phosphorylation landscape of Tau protein across *APOE* genotypes. Tau-phosphorylation sites were identified by mass spectrometry in anti-pTau PHF1 immunoprecipitated fractions (*n* = 5 *APOE*^ε3/ε3^ cases, *n* = 5 *APOE*^ε4/ε4^ cases). All detected sites were mapped along the Tau-protein sequence, based on the 2N4R Tau isoform of 441 amino acids. The relative abundance of each phosphorylation site was represented by the node color, as detailed in the legend. The node color-code reflects the average percentage of phosphorylated peptide normalized to all observed versions of the respective peptide, for each *APOE* group. Note that some phosphorylation sites were detected across multiple Trypsin cleavage products, hence the use of summed percentages for all peptides containing the same site(s). The frequency of observation of each phosphorylated modification within a group is represented separately by the node size, as shown in the legend. The node size reflects the number of cases out of 5 total presenting with the corresponding phosphorylation site(s), for each *APOE* group. The absence of any phosphorylation site is represented by a “- “

The MS analysis of IP_PHF1_ products identified 30 phosphorylation sites associated with Tau proteins. Each site was mapped along Tau sequence based on the 2N4R Tau isoform of 441 amino acids, associated with its relative abundance and frequency of observation within each *APOE* group. MS analysis could not always identify the exact position of a phosphate group between T403 and S404 as the peptide backbone cleavage between these two amino acids was sometimes missing and because of an identical retention time under the LC conditions used. We therefore combined the information associated with the pT403 and pS404 sites in this analysis, referred to as pT403-pS404. The most abundant and frequently detected phosphorylation sites, alone or in combination on the same peptide, were the following: pT175 with pT181, pT181, pS202, pT212 with pT217, pT217, pT231, pT231 with pS235, pS262, pS289, pS396, pS396 with pS400, pS396 with pT403-pS404, pT403-pS404. Of note, the phosphorylation sites recognized by the anti-pTau PHF1 antibody, pS396 and pS404, were observed for all cases in both *APOE* groups, validating our success in enriching the targeted pTau proteins. As shown in Fig. 4 and Online Resource 4, the phosphorylation landscapes of Tau protein analyzed in our samples were similar between *APOE*^ε3/ε3^ and *APOE*^ε4/ε4^ groups, except for one observation: the combination of both pT175 and pT181 sites was only seen in the *APOE*^ε4/ε4^ group, in which it was observed in three out of five cases on a unique peptide (IPAKpTPPAPKpTPPSSGEPPK). The unphosphorylated version of this peptide was not identified, which confirms this modification further as Trypsin can only cleave peptide bonds at the C-terminal side of lysine (K) and arginine (R) residues and will not cleave at the N-terminal side of a phosphorylated residue (Fig. 4 and Online Resource 4).

### Characterization of Tau lesions among APOE groups

**Fig. 5:**
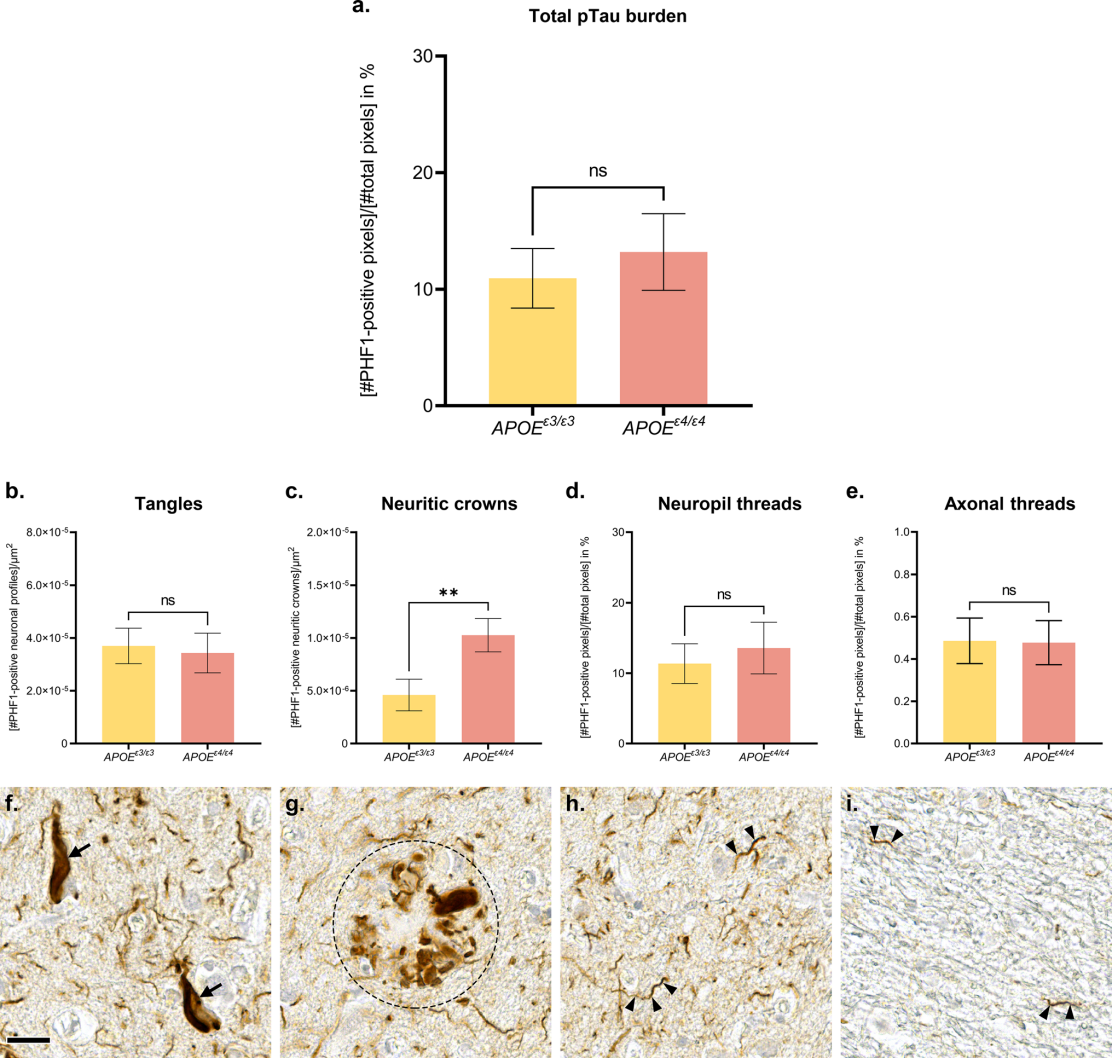
Influence of *APOE*^ε4^ on Tau pathology in the frontal cortex of advanced AD cases. **a**–**i**. Histologic analysis of Tau pathology conducted on a total of *n* = 11 *APOE*^ε3/ε3^ cases and *n* = 10 *APOE*^ε4/ε4^ cases, presenting with advanced AD. Anti-pTau PHF1 immunohistochemistry with DAB revelation. Human brain, formalin-fixed paraffin-embedded frontal cortex, × 40 magnification. **a** Quantification of the total pTau burden in the frontal cortex as an averaged percentage of PHF1 positive pixels in *n* = 3 ROIs/case of 5.0 × 10^6^ µm^2^ (± 0.5 × 10^6^ µm^2^) each, encompassing all cortical layers and evenly spread over the cortical section. **b** Quantification of the density of tangles in the frontal cortex as a number of PHF1 positive neuronal profiles (pre-tangles, tangles, ghost tangles) per μm^2^. Analysis performed on *n* = 1 ROI/case of 5.0 × 10^6^ µm^2^ (± 0.5 × 10^6^ µm^2^) covering all the cortical layers of the grey matter. **c** Quantification of the density of neuritic crowns in the frontal cortex as a number of PHF1 positive neuritic crowns per μm^2^. Analysis performed on *n* = 1 ROI/case of 5.0 × 10^6^ µm^2^ (± 0.5 × 10^6^ µm^2^) covering all the cortical layers of the grey matter. **d** Quantification of the burden of PHF1 positive neuropil threads as an averaged percentage of PHF1 positive pixels from *n* = 10 sub-ROIs/case of 1.0 × 10^4^ µm^2^, covering neuropil areas evenly spread over all cortical layers of the grey matter and devoid of any neuronal profiles or neuritic crowns, within *n* = 1 ROI/case of 5.0 × 10^6^ µm^2^ (± 0.5 × 10^6^ µm^2^). e. Quantification of the burden of PHF1 positive axonal threads as an averaged percentage of PHF1 positive pixels from *n* = 10 ROIs/case of 1.0 × 10^5^ µm^2^ covering the white matter. **f**–**h**. Illustration of a tangle (f, arrows), neuritic crown (**g**, circle) and neuropil thread (**h**, arrowheads) observed in the grey matter. **i** Illustration of thin and fragmented axonal threads seen in the white matter (arrowheads). Scale bar: 20 μm. Unpaired non-parametric Mann–Whitney tests, ns *p* > 0.05, **p < 0.01. *AD *Alzheimer’s disease,* ROI *region of interest

Our proteomic observations suggest that the expression of the AD risk factor *APOE*^ε4^ mostly impacts pTau-subcellular location. To validate this hypothesis, a comparative quantification of Tau pathology across *APOE* genotypes was performed in the frontal cortex of 21 advanced sporadic AD cases, after an anti-pTau PHF1 immunohistochemistry (*n* = 11 *APOE*^ε3/ε3^ and *n* = 10 *APOE*^*ε4/ε4*^ cases). As expected, the pTau burden detected in the grey matter of the frontal cortex was similar between the *APOE*^ε3/ε3^ and *APOE*^*ε4/ε4*^ groups, composed of advanced AD cases (unpaired non-parametric Mann–Whitney test, *p* > 0.05; Fig. 5a). To assess the influence of *APOE*^*ε4*^ on pTau-subcellular location, the distribution of pTau aggregates among the various neuronal compartment were further analyzed. All of the well-characterized subtypes of pTau pathologic lesions linked to AD were observed in both *APOE*^ε3/ε3^ and *APOE*^*ε4/ε4*^ groups, including: neurofibrillary tangles consisting of pTau aggregates in the neuronal soma (“tangles”, Fig. 5b and f), pTau-positive neuritic crowns consisting of degenerated axonal terminals and enlarged synapses which compose the neuritic amyloid plaques (“neuritic crowns”, Fig. 5c and g), neuropil threads consisting of the accumulation of pTau mostly in dendrites (“neuropil threads”, Fig. 5d and h), and axonal threads consisting of thin and fragmented threads observed in the white matter (“axonal threads”, Fig. 5e and i). The quantifications of tangles, neuropil threads and axonal threads did not show any differences between the *APOE*^ε3/ε3^ and *APOE*^*ε4/ε4*^ groups (unpaired non-parametric Mann–Whitney tests, *p* > 0.05, Fig. 5b, d–e). The density of pTau-positive neuritic crowns was, however, significantly higher in the *APOE*^*ε4/ε4*^ cases, in comparison with the *APOE*^*ε3/ε3*^ cases (unpaired non-parametric Mann–Whitney test, *p* = 0.0062, Fig. 5c); results were similar after data normalization with the pTau burden of each respective ROI (unpaired non-parametric Mann–Whitney test, *p* = 0.0159; not shown).

### CAA profile among APOE groups

**Fig. 6:**
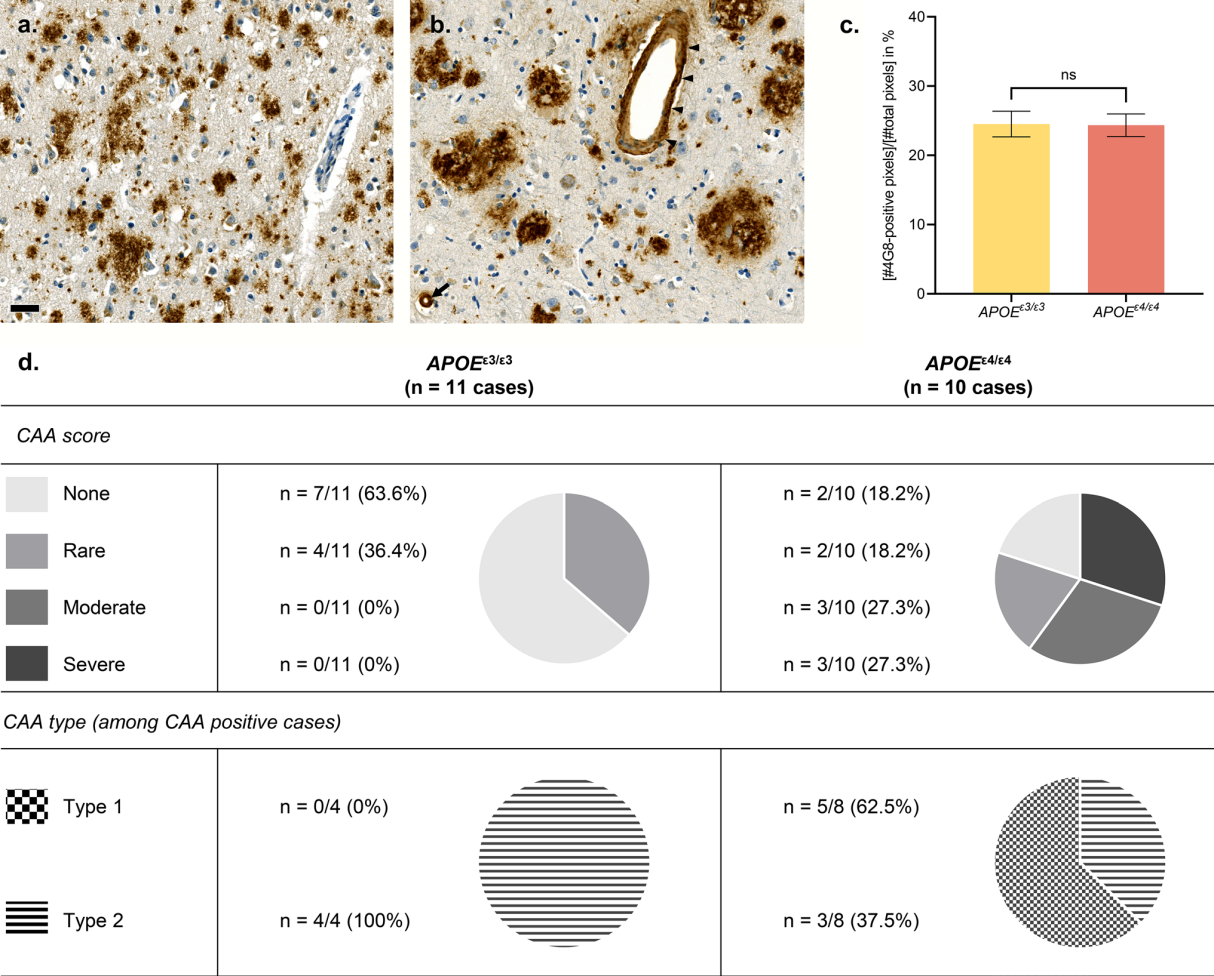
Influence of *APOE*^ε4^ on CAA frequency, severity and type in the frontal cortex of advanced AD cases. **a**–**d**. Histologic analysis of Aβ pathology conducted on a total of *n* = 11 *APOE*^ε3/ε3^ cases and *n* = 10 *APOE*^ε4/ε4^ cases, presenting with advanced AD. Anti-Aβ 4G8 immunohistochemistry with DAB revelation. Human brain, formalin-fixed paraffin-embedded frontal cortex, × 20 magnification. **a**–**b** Representative illustration of Aβ deposits seen in the frontal cortex of an *APOE*^ε3/ε3^ case (**a**) and *APOE*^ε4/ε4^ case (**b**). Note the presence of CAA in the parenchyma of the *APOE*^ε4/ε4^ case, involving a capillary (arrow) and a larger vessel (arrowheads). Scale bar: 20 μm. **c** Quantification of the total Aβ burden in the frontal cortex as an averaged percentage of 4G8 positive pixels in *n* = 3 ROIs/case of 5.0 × 10^6^ µm^2^ (± 0.5 × 10^6^ µm^2^) each, encompassing all cortical layers and evenly spread over the cortical section. Unpaired non-parametric Mann–Whitney tests, ns *p* > 0.05. **d** Descriptive table and circle diagrams showing the frequency, severity and type of CAA among a total of *n* = 11 *APOE*^ε3/ε3^ cases and *n* = 10 *APOE*^ε4/ε4^ cases. A semi-quantitative CAA score was attributed to each case as follows: none (no Aβ-positive vessel detected), rare (scattered Aβ-positive vessels), moderate (about 50% of Aβ-positive vessels), severe (about 100% of Aβ-positive vessels). The CAA type was evaluated among CAA positive cases as follows: type 1 (capillary CAA associated with CAA in larger vessels) or type 2 (CAA without capillary involvement). *AD* Alzheimer’s disease, *CAA* Cerebral Amyloid Angiopathy, *ROI* region of interest

A comparative analysis of Aβ pathology across *APOE* genotypes was performed in the frontal cortex of 21 advanced sporadic AD cases, after an anti-Aβ 4G8 immunohistochemistry (*n* = 11 *APOE*^ε3/ε3^ and *n *= 10 *APOE*^*ε4/ε4*^ cases, Fig. 6a–b). The Aβ burden detected in the grey matter of the frontal cortex was similar between the *APOE*^ε3/ε3^ and *APOE*^*ε4/ε4*^ groups, composed of advanced AD cases (unpaired non-parametric Mann–Whitney test, *p* > 0.05; Fig. 6c). It has been recently suggested that CAA interacts with neuritic plaques to enhance Tau pathology [65]. As the expression of ApoE4 is known to exacerbate CAA [71], in addition of being strongly associated with the presence of capillary CAA [80, 81], the CAA profile was analyzed in the parenchyma of the frontal cortex in our cohort (Fig. 6d). A semi-quantitative score was attributed to each case as follows: “none” when no Aβ-positive vessel was detected in the cortical parenchyma, “rare” when only a few scattered Aβ-positive vessels were seen, “moderate” if about 50% of vessels were Aβ-positive, “severe” if about 100% of vessels were Aβ-positive. The presence of CAA was detected in 36.4% of cases in the *APOE*^ε3/ε3^ group, while this proportion reached 72.8% of cases in the *APOE*^ε4/ε4^ group. When present, CAA was scored as “rare” in 100% of the CAA positive cases in the *APOE*^ε3/ε3^ group. In contrast, the CAA scores observed in the *APOE*^ε4/ε4^ group were either “rare” (25% of the CAA positive cases), “moderate” (37.5% of the CAA positive cases) or “severe” (37.5% of the CAA positive cases; Fig. 6). The distribution of CAA types was different among *APOE* groups: whereas no capillary CAA was observed among the CAA positive cases of the *APOE*^ε3/ε3^ group (0% of CAA type 1, 100% of CAA type 2), the presence of capillary CAA was predominant among the CAA positive cases of the *APOE*^ε4/ε4^ group (62.5% of CAA type 1, 37.5% of CAA type 2).

## Discussion

By profiling the pTau interactome selectively in *APOE*^*ε3/ε3*^ and *APOE*^*ε4/ε4*^ carriers for the first time, we discovered that the *APOE* genotype significantly influences the pTau pS396/pS404 interactome. We determined that the pTau interactome reflects a different subcellular localization of pTau aggregates in *APOE*^*ε3/ε3*^ and *APOE*^*ε4/ε4*^ cases. We confirmed this result through our follow-up immunohistochemistry studies and propose that the AD risk factor *APOE*^ε4^ facilitates Tau-pathology progression by enhancing the accumulation of pTau in axonal endings and synapses, particularly in Aβ-affected brain regions.

A total of 80 and 68 proteins were identified as probable pTau interactors in the *APOE*^*ε3/ε3*^ and *APOE*^*ε4/ε4*^ groups, including 33 proteins that interacted with pTau irrespective of *APOE* genotype. These 33 common pTau interactors showed a preserved interaction of pTau with proteins involved in the ubiquitin–proteasome system among *APOE*^*ε3/ε3*^ and *APOE*^*ε4/ε4*^ cases. This observation confirms the importance of the ubiquitin–proteasome system in a context of defective protein clearance and protein accumulation [56, 61], while highlighting some of its key members consistently observed among pTau interactors in the AD brain, such as: SQSTM1 (also known as p62), which is involved in the shuttle of polyubiquitinated Tau for proteasomal degradation; ubiquitin/polyubiquitin precursors such as RPS27A and UBC; ubiquitin protein ligases such as ARMC8 and UBR4; several members of the PSMC and PSMD families constituting the proteasome [5, 10, 26, 60]. The detected PSMC and PSMD members constitute only the lid and base of the 26S proteasome regulatory subunit involved in substrate recognition, deubiquitylation and unfolding, underlining the specificity of our approach [7]. Proteins involved in the regulation of RNA processing were also detected among the common pTau interactors, supporting an important role of RNA metabolism and translational stress response in Tau pathology as suggested by other studies [43]. These new results extend our previous analysis by bringing to light additional RNA-associated proteins of interest, which were previously detected as present in the neurofibrillary tangle proteome, although they were below the threshold to be considered as pTau interactors so far [26]: the splicing factors SRSF1 (also known as SF2/ASF) and TRA2B, involved in the regulation of the alternative splicing of Tau exon 10 impacting the ratio of Tau isoforms with three or four microtubule binding repeats domains (3R or 4R, with a 4R:3R ratio shifted from approximately 1:1 to 2:1 in AD) [17, 20, 33]. Interestingly, a large proportion of the proteins identified as probable pTau interactors in the *APOE*^ε3/ε3^ and *APOE*^*ε4/ε4*^ groups did not overlap. This observation cannot be explained by the inclusion of differing stages of AD pathology among *APOE* groups, as this study was conducted on an homogenous selection of sporadic cases diagnosed with an advanced AD pathology at autopsy (A3, B3, C3 scores [55]). This key result supports an influence of *APOE* expression on pTau metabolism in the AD brain.

A total of 47 proteins were identified as probable pTau interactors only for *APOE*^ε3/ε3^ cases, while 35 proteins were detected as probable pTau interactors only for *APOE*^ε4/ε4^ cases. A robust segregation of their associated functions and cell compartments was observed. The pTau interactome specific to *APOE*^ε3/ε3^ cases contained a majority of nucleoplasmic proteins, involved in RNA binding and processing. PNN, a newly identified pTau interactor, was particularly enriched in *APOE*^ε3/ε3^ cases and recently characterized in vitro as involved in the formation of RNA condensates defining subcellular sites of Tau aggregation [45, 46]. In vitro, Tau coacervates with polyanions, such as RNA, into liquid droplets [38, 47, 95]. The colocalization of Tau with RNA and RNA binding proteins in the cell can result in the formation of stress granules involved in protein-level regulation [2, 62], although experimental observations suggest that such complexes drive Tau aggregation [2, 38, 42, 52]. In AD neurons, pTau is accumulated at the nuclear envelope in a discrete manner, which correlates with nuclear component mis-localization [29, 38]. Our results support a predominance of these biologic events in *APOE*^ε3/ε3^ cases. They can be protective in the early stages of the pathology, slowing down Tau pathology by sequestering pTau species at the nucleus while triggering the translational stress response. Over time, deleterious outcomes may take over including a promotion of pTau self-aggregation [2, 38, 42, 52] or a nucleocytoplasmic transport disruption [29, 94]. In contrast, the pTau interactome specific to *APOE*^ε4/ε4^ cases contained a majority of synaptic proteins, involved in cell transport. ARRB1, a newly identified pTau interactor particularly enriched in the *APOE*^ε4/ε4^ group, is a representative synaptic protein recently described as a promoter of Tau pathology [92]. Tau pathology in *APOE*^ε4/ε4^ cases may be characterized by a predominant pool of dynamic pTau species, more prone to be transported to the synapse, which can facilitate the trans-synaptic progression of Tau pathology [88, 93] and Tau-mediated synaptic disruption [86]. We did not observe any major effect of *APOE*^ε4^ on Tau-phosphorylation sites, as suggested by recent experimental observations [69]. Only the pT175/pT181 modification was specific to the *APOE*^ε4/ε4^ group, although this result requires further investigations since this study is based on a selected fraction of pTau that is phosphorylated on pS396/pS404 and obtained from *post-mortem* material [32]. We suggest that the expression of the AD risk factor *APOE*^ε4^ mostly impacts pTau-subcellular location.

A comprehensive analysis of the various subcellular lesions composing Tau pathology in *APOE*^ε3/ε3^ and *APOE*^*ε4/ε4*^ cases confirmed our hypothesis. The density of pTau-positive neuritic crowns was higher in *APOE*^ε4/ε4^ vs *APOE*^ε3/ε3^ cases, despite the confirmation of an even burden of Tau pathology among cases. Neuritic crowns are made of pTau-positive degenerated neurites wrapping the most mature type of Aβ deposits, constituting the neuritic amyloid plaque – or senile plaque [27]. The immunohistochemical signature of these pTau-positive neuritic crowns [72, 84], along with the observation of presynaptic vesicles [79], demonstrate their axonal nature. This observation aligns with our proteomics findings and further support an effect of *APOE*^ε4^ on pTau cellular transport and relocation toward axonal endings and synapses. Tau pathology progresses from neuron to neuron through synaptic connections [16, 28, 83, 88, 93]; we hypothesize that the spreading of pathological pTau species is accelerated in *APOE*^ε4/ε4^ carriers, in accordance with recent clinical observations [8, 75]. In vivo experiments support an Aβ-independent influence of *APOE*^*ε4*^ on Tau-pathology spreading, by demonstrating an exacerbation of Tau pathology in PS19 mice expressing human A*POE*^ε4/ε4^ [74], but a recent study questions this scenario [21]. These results suggest a direct consequence of *APOE* genotype itself on Tau pathology (*e.g.,* the proteins identified in the present study as pTau interactors more associated with one *APOE* genotype or another could have different expression levels in control brains). This possibility is illustrated by the recent identification of 25 unique proteins defining the incipient AD proteomic signature, including 24 increased in young heterogenous *APOE*^*ε4*^ carriers, while 1 protein was reduced in comparison to aged-matched *APOEε4* non-carriers [67]. Although we could not identify any of our 47 *APOE*^*ε3*^-associated and 35 *APOE*^*ε4*^-associated pTau interactors in this list of 25 candidates, future studies are needed to address this possibility by better understanding how the AD risk factor *APOE*^ε4^ shapes the basal metabolism of the brain. While they are not mutually exclusive, an alternative scenario involves an Aβ-mediated effect of *APOE* on Tau pathology. The expression of the *APOE*^ε4^ allele is indeed strongly associated with an exacerbation of Aβ pathology, by promoting particularly the development of neuritic amyloid plaques and of CAA with a capillary involvement [70, 81, 90], as confirmed in our cohort. Recent neuropathological and clinical studies show that CAA interacts with neuritic amyloid plaques to enhance tau pathology and white-matter rarefaction [50, 59, 65]. We propose that the AD risk factor *APOE*^ε4^ promotes neuritic degeneration, resulting in the accumulation of pTau in axonal endings and synapses which may facilitate Tau-pathology progression, particularly toward Aβ-affected brain regions.

## Limitations

While there were many consistencies with our previous pTau interactome study [26], our results did not completely replicate our previous findings. The inter-individual variability associated with *post-mortem* human brain studies, combined with a modified MS method used here, could explain these differences. In our current study, our MS protocol was adjusted to simplify our workflow. To prevent the excess of antibodies co-eluted in the immunoprecipitated product from hindering the detection of proteins with similar mass weight and elution times, immunoprecipitated products were previously run on a gel from which bands containing antibodies were excised and analyzed separately on the mass spectrometer [26]. Here, we opted for a different strategy to minimize sample processing prior to MS analysis: a proteolytic digestion was performed straight on the antigen–antibody-bead complexes without removing antibodies prior to downstream proteomic analysis, increasing the power of our study by allowing a better technical consistency. Furthermore, our designation of “pTau interactors” relied on the binarization of a continuum of probabilistic scores, based on the use of a stringent threshold corresponding to a FDR of 5%. Although this strategy allows for the reduction of false positives and a focus on the most probable pTau interactors for a more stringent analysis, it increases the risk of false negatives among the proteins that did not pass the threshold. Altogether, these limitations explain why some important pTau interactors may be missing in the present study and emphasize the need to generate more AD-related proteomic datasets, to counterbalance inter-experimental differences. Although pathologically relevant, these proteomic findings may reflect advanced biologic responses in AD, as they are associated with the late epitope pTau pS396/pS404 extracted from advanced AD cases. It is still unclear if a different subset of pTau species interacts with different proteins. These aspects need to be further addressed in future investigations by exploring the interactome of alternative pTau epitopes, especially early ones extracted from early and intermediate stage AD cases.

## Conclusion and perspectives

This study provides evidence for an influence of *APOE* expression on pTau-subcellular location, suggesting a greater variation of Tau pathology across AD cases. Indeed, these new results emphasize the complexity of Tau studies, as factors such as genotype can modify the subcellular localization of pTau and therefore its interactome. Our results pave the way to the potential identification of new therapeutic targets specific for *APOE*^ε4^ carriers.

### Supplementary Information

Below is the link to the electronic supplementary material.

Supplementary file1 (XLSX 678 KB)

Supplementary file2 (XLSX 407 KB)

Supplementary file3 (XLSX 21 KB)

Supplementary file4 (XLSX 129 KB)

## Data Availability

The mass spectrometric raw files are accessible at https://massive.ucsd.edu under accession MassIVE MSV000094757 and at www.proteomexchange.org under accession PXD052263.
